# The epidemiology of multiple primary cancers in Belgium (2004–2017): Incidence, proportion, risk, stage and impact on relative survival estimates

**DOI:** 10.1186/s12885-023-10777-7

**Published:** 2023-04-17

**Authors:** Gilles Macq, Geert Silversmit, Freija Verdoodt, Liesbet Van Eycken

**Affiliations:** Belgian Cancer Registry, Rue Royale 215, box 7 1210 Brussels, Belgium

**Keywords:** Cancer, Cancer incidence, Multiple primary cancer diagnosis, Relative survival, Standardised incidence ratio, Cancer stage

## Abstract

**Background:**

As both life expectancy and cancer survival improve, the incidence of multiple primary cancer has augmented and is expected to further increase. This study describes for the first time the epidemiology of multiple invasive tumours in Belgium.

**Methods:**

This nationwide study, based on all cancers diagnosed between 2004 and 2017 in Belgium, describes the proportion of multiple primary cancer, its evolution over time, the impact of inclusion or exclusion of multiple primary cancer on relative survival estimates, the risk of developing a second primary cancer, and the difference in stage between first and second primary cancer for the same patient.

**Results:**

The proportion of multiple primary cancer increases with age, varies across cancer sites (from 4% for testis cancer to 22.8% for oesophageal cancer), is higher in men than in women, and has linearly increased over time. The inclusion of multiple primary cancer resulted in smaller 5-year relative survival and this impact is more pronounced in cancer sites with high relative survival. Patients with a first primary cancer have an increased risk to develop a new primary cancer compared to the population without a previous cancer history (1.27 and 1.59 times higher in men and women, respectively) and this risk depends on cancer site. Second primary cancers are associated with more advanced stages and more unknown stages than the corresponding first cancer diagnosis.

**Conclusions:**

This study describes multiple primary cancer according to several measures (proportion, standardised incidence ratio for an second primary cancer, impact of multiple primary cancer on relative survival and differences according to stage) for the first time in Belgium. The results are based on data of a population-based cancer registry with a relatively recent onset (2004).

**Supplementary Information:**

The online version contains supplementary material available at 10.1186/s12885-023-10777-7.

## Background

The occurrence of a second or subsequent primary cancer (i.e., multiple cancer diagnosis) has augmented and is expected to continue to increase because cancer survival has improved over the past decades [1, 2]. While the risk of developing a second primary cancer (SPC) has been calculated in several countries [3–7], it has never been evaluated at the population level in Belgium. Such information is, however, crucial for the follow-up of cancer patients, for example to identify sites at risk for a SPC after a first primary cancer (FPC).

Only recently, studies started to consider the impact of multiple primary cancers (MPC) on relative survival (RS) [8–10]. RS gives an estimate of the net survival probability for patients, which is the probability of survival in a hypothetical situation where the cancer is the only possible cause of death [11, 12]. The probability that a previously diagnosed cancer of the same patient has been registered depends on how long the registry has been in operation: the probability is greater in ‘older’ than in ‘younger’ registries [8]. As a consequence, it is possible that second or subsequent primary cancer cases are not recognized as such and are thus included in survival analysis as if they were FPC. This implies that omitting MPC from survival estimates could lead to biased comparisons over time even within one cancer registry. In this nationwide study, we evaluated MPC, using data from the Belgian Cancer Registry, which is considered among the ‘younger’ population-based cancer registries in Europe.

According to the Belgian legislation, cancer registration is mandatory since 2003 and full population coverage of cancer registration is considered since 2004. The database of the Belgian Cancer Registry is considered as more than 95% complete [13]. Before the Belgian Cancer Registry was established, some initiatives took place in Belgium. In 1983, the National Cancer Registry was founded where data were obtained from Belgian health insurance companies. Some companies started cancer registration in the early fourties, however, evaluation showed a considerable underregistration [14]. With the aim of rectifying this underregistration, in the northern region of Belgium, Flanders, the Flemish cancer registry was created in 1994. Registration started in 1997 and the methodology evolved over the years 1997 to 1999. Registration was quasi complete from 1999–2000 for Flanders. In this study, the evolution of the proportion of MPC is presented over a period of a 14-year incidence period from the start of registration at the Belgian Cancer Registry in 2004.

## Methods

### Nationwide cohort study

Information on cancer diagnoses was obtained from the Belgian Cancer Registry. Residents of Belgium, aged 15 years or older at diagnosis, with a known national social security number and diagnosed with at least one primary malignant tumour other than non-melanoma skin cancer in the period 2004–2017 were considered. Primary cancers with an uncertain date of diagnosis, with a date of diagnosis equal to the date of death or loss-to-follow-up of the patient, and diagnosed before 2004 or after 2017 were excluded (corresponding to 3,454 excluded cancers, or 0.4% of all primary cancers other than non-melanoma skin cancer in our population of interest). Cancers diagnosed before 2004 were not included for the analyses, however registered cancers before 2004 for patients included in this study have been used to define the MPC status of cancers included in this study. Cancer cases were classified based on the International Classification of Diseases for Oncology, Third Edition (ICD-O-3) [15]. We applied coding rules based on the IARC (International Agency for Research on Cancer) recommendations [16] to distinguish between a FPC and a subsequent primary cancer. According to those rules, a primary cancer is one that originates in a primary site or tissue and is not an extension, nor a recurrence, nor a metastasis. Based on these rules, the following information must be considered to distinguish a MPC from an extension, recurrence or metastasis: topography, laterality, histology and behaviour. There are rules for each criterion (for more information, see Supplementary file 1).

In the case of several cancers diagnosed on the same day, the cancers were classified according to their stage (i.e., prioritizing pathological stage over clinical stage, except when there is clinical proof of distant metastasis), with the highest stage considered as first (IV > III > II > I > Diagnoses with an unknown stage > Diagnoses with a histological diagnosis where no stage can be evaluated > 0). If several cancers diagnosed on one day had the same stage, then the order between them was random, depending on the order in the dataset.

In this study, MPC have been analysed at the tumour and patient level. Three analyses have been performed on the tumour level: the proportion of MPC according to age, gender, region and cancer site, its evolution in time and the impact of MPC on RS estimates. Two other analyses have been conducted on the patient level: the risk of developing an SPC and the difference in stage between FPC and SPC.

### Proportion of MPC

Trends over time of the proportion of MPC were evaluated with linear models ($$p={\beta }_{0}+{\beta }_{1}\times (year of incidence)$$). In this kind of model, $${\beta }_{1}$$ corresponds to the average increase in the proportion *p* of MPC by year. Regression models have been built for each combination of gender and age category (15–44, 45–54, 55–64, 65–74 and ≥ 75 years), and for each gender and all age categories combined.

### Impact of MPC on RS estimate

RS is defined as the ratio of the observed survival and the survival that would have been expected within a similar cohort from the general population, matched on age, sex, region and year of diagnosis. The Ederer II method was used to calculate expected survival [17]. RS analyses were based on a publicly available algorithm [18], using time intervals of 1 year wide. In addition, age-standardised RS estimates were computed using the standard cancer populations proposed by Corazziari et al. [19]. Survival time was calculated from the date of diagnosis until the date of death or until the date with the last known vital status derived from the Belgian Crossroads Bank for Social Security on July 1, 2018.

The RS was computed once for FPC cases only, and once for all cancers; so including first and subsequent primary cancers cases. The differences between the two data selection procedures were assessed relative to the value of the RS calculated on FPC only, stratified by cancer site and gender. Simple linear regression has been applied on this absolute difference in RS estimates over cancer type for men and women separately.

### Standardised incidence ratio (risk of developing a SPC)

The standardised incidence ratio (SIR) of a SPC after an FPC, was calculated as the ratio between the observed number of SPC cases and an expected number of SPC cases. The former corresponds to the number of SPC observed in the population with a FPC. The latter corresponds to the number of SPC that would have been observed if the FPC incidence rates observed in the cancer-free population are applied to the patients with a FPC. The incidence rates in the population free of cancer have been calculated by gender, cancer site, region, age category (in 5 year age categories) and incidence year. To obtain the number of expected cases, these incidences rates have been multiplied by the corresponding sum of person years in the study population of patients with one FPC diagnosis. The person time was computed as the time since the date of diagnosis of the FPC to one of the following dates: (i) date of death, (ii) date of diagnosis of the SPC, or (iii) last date of follow up if it’s before the end of 2017, or (iv) December 31, 2017 if the last date of follow up comes after this date, whichever came first. If the SIR is higher (lower) than 1, the risk of developing a SPC is higher (lower) than the risk of developing a FPC in the Belgian population free of cancer. Confidence intervals on the SIR have been calculated by using the exact confidence interval for a Poisson variable [20, 21].

### Difference in stage distribution between FPC and SPC

The difference in stage between FPC and SPC has been studied taking 5 patterns into account: an identical stage, an increase in stage, a decrease, a known stage for the FPC and an unknown stage for the SPC and an unknown stage for the FPC and a known stage for the SPC. Proportions and confidence interval of these differences have been calculated according to gender and type of cancer. Confidence intervals have been computed based on exact binomial quantiles. Cancers diagnosed at the same day have been excluded for this analysis as the rule used to order cancers diagnosed the same day is based on stage.

A significance level of 5% was applied for all statistical tests and 95% CI are reported. Statistical analyses were performed by using SAS 9.4 (SAS institute, Cary, NC). Figures were created with SAS version 9.4 or R version 3.6.1.

## Results

A total of 809,081 patients representing 885,718 primary cancers diagnosed in 2004–2017 were included in this study. About 90% of the MPCs are a SPC and 9% are third primary cancer. Fourth or higher primary cancer represents less than 1% of the MPCs. The database contains 67,344 (8.3%) patients with at least a FPC and a SPC. Among these patients, 5,804 (8.6% of 67,344) had a FPC and SPC diagnosed on the same day. Moreover, 4,079 of these patients (70,2% of 5,804) have the same cancer site for their FPC and SPC. About three quarters of them are women with breast cancers (80.3%) and colorectal cancers (13.7%) while in men, these are primarily colorectal cancers (63.2%) and head and neck cancers (9.6%).

### Proportion of MPC

The proportion of MPC by gender, age, region and cancer type is given in Table 1. For all tumours combined, the proportion of MPC was 12.2% during the entire incidence period 2004–2017. The proportion of MPC was 7.3% in 2004 and increased to 15.2% in 2017. The proportion of MPC was higher in men (13.2%) than in women (11.2%) and increased with age from 3.6% for the youngest patients (15–44 years) to 16% for patients older than 74 years. In terms of cancer sites, a large variability in proportion of MPC was observed. The proportion of MPC was highest for oesophagus (17.7%), kidney (17.2%) and bladder (15.8%), and lowest for Hodgkin’s lymphoma (5.5%), cervix (4.8%) and testis (3.4%). The largest increase in proportion of MPC from 2004 to 2017 was observed for bone and soft tissues (+ 11.4 percentage point), pancreas (+ 11.2 percentage point), oesophagus and head and neck (+ 11.1 percentage point﻿). The median time from first to second primary cancer registration among the 67,344 patients with at least a FPC and a SPC registered is summarised and visualised in Supplementary file 2. In general, median time is shortest for FPC sites with low survival, like pancreas and oesophagus, and largest for FPC sites with good survival like thyroid and prostate. Median time for the same FPC site can depend strongly on the SPC site.

**Table 1:** Number of primary cancers (N), Proportion of multiple primary cancer (MCP) in 2004 [a], in 2017 [b] and the difference in percentage point between the proportion of MPC in 2017 and the proportion of MPC in 2004 ([b] -[a]) by gender, age category, region and cancer type

		Men and women					Men					Women				
		Period 2004–2017	% MPC by year of incidence				Period 2004–2017	% MPC by year of incidence				Period 2004–2017	% MPC by year of incidence			
		N	% MPC	2004 (*N* = 57,818) [a]	2017 (*N* = 68,965) [b]	[b]-[a]	N	% MPC	2004 (*N* = 31,763) [a]	2017 (*N* = 36,670) [b]	[b]-[a]	N	% MPC	2004 (*N* = 26,055) [a]	2017 (*N* = 32,295) [b]	[b]-[a]
All cancers		885,718	12.2	7.3	15.2	7.9	–	–	–	–	–	–	–	–	–	–
Gender	Men	472,390	13.2	7.9	16.4	8.4	–	–	–	–	–	–	–	–	–	–
	Women	413,328	11.1	6.6	14.0	7.3	–	–	–	–	–	–	–	–	–	–
Age	15–44 years	59,832	3.6	2.5	3.9	1.4	20,713	3.1	2.0	3.8	1.9	39,119	3.8	2.7	3.9	1.2
	45–54 years	104,821	6.7	5.2	7.3	2.1	42,029	5.9	4.7	6.2	1.5	62,792	7.3	5.5	8.0	2.5
	55–64 years	196,881	10.0	6.5	12.1	5.6	108,549	9.4	5.8	11.0	5.2	88,332	10.9	7.5	13.4	5.9
	65–74 years	247,706	14.1	8.5	17.3	8.8	151,182	14.4	9.0	17.4	8.3	96,524	13.7	7.6	17.1	9.5
	+ 75 years	276,478	16.0	8.8	20.5	11.7	149,917	18.3	10.1	23.2	13.1	126,561	13.3	7.1	17.3	10.2
Region	Brussels capital	71,033	11.2	6.2	14.1	7.8	34,264	11.9	6.5	14.7	8.3	36,769	10.5	6.0	13.4	7.4
	Flanders	529,926	12.6	7.9	15.4	7.5	289,862	13.7	8.6	16.7	8.1	240,064	11.3	7.1	14.0	6.9
	Wallonia	284,759	11.7	6.6	15.1	8.6	148,264	12.5	7.1	16.2	9.1	136,495	10.9	6.0	14.1	8.1
Cancer type	Oesophagus	13,306	17.7	11.6	22.8	11.1	9,793	18.1	11.1	20.9	9.9	3,513	16.7	13.1	27.6	14.5
	Kidney	21,679	17.2	12.6	20.4	7.8	13,723	18.5	13.1	21.4	8.3	7,956	14.9	11.8	18.5	6.7
	Bladder	31,177	15.8	9.8	18.7	8.9	24,705	16.3	10.5	18.3	7.8	6,472	14.0	6.9	20.1	13.2
	Lung	108,595	15.8	9.5	19.0	9.6	77,767	16.2	9.7	19.9	10.2	30,828	14.7	8.7	17.3	8.6
	Head and neck	35,835	14.1	8.5	19.5	11.1	27,044	14.3	8.8	20.1	11.3	8,791	13.6	7.2	17.8	10.6
	Bone and soft tissues	5,948	13.8	8.2	19.6	11.4	3,231	13.4	8.0	17.5	9.5	2,717	14.3	8.5	22.2	13.7
	Colorectal	116,110	13.8	8.0	16.5	8.5	64,150	15.2	9.0	18.1	9.1	51,960	12.1	6.9	14.6	7.7
	Stomach	20,081	13.7	8.1	17.9	9.8	12,556	14.6	9.7	18.6	8.9	7,525	12.1	5.2	16.8	11.6
	Liver	9,792	13.4	6.4	14.4	8.0	6,897	14.4	6.5	13.2	6.7	2,895	11.2	6.4	17.4	11.0
	Pancreas	20,311	13.0	6.9	18.2	11.2	10,427	14.3	8.5	20.2	11.7	9,884	11.7	5.1	16.1	11.0
	Leukaemia	21,883	12.8	7.8	17.0	9.3	12,612	13.2	7.8	16.8	9.0	9,271	12.3	7.7	17.4	9.7
	Non-Hodgkin’s lymphoma	26,439	11.7	7.0	15.1	8.0	14,412	12.8	6.8	16.5	9.7	12,027	10.3	7.4	13.3	5.9
	Multiple myeloma	10,580	10.9	6.9	15.9	9.0	5,814	12.4	7.8	16.9	9.1	4,766	9.0	6.0	14.6	8.6
	Melanoma of skin	31,618	10.6	6.5	13.6	7.1	13,071	12.3	7.8	15.1	7.3	18,547	9.4	5.5	12.5	7.0
	Ovary	11,666	10.5	8.8	11.8	3.0	0	–	–	–	–	11,666	10.5	8.8	11.8	3.0
	Corpus uteri	19,828	10.3	7.2	13.4	6.2	0	–	–	–	–	19,828	10.3	7.2	13.4	6.2
	Female Breast	149,530	10.1	6.0	12.2	6.2	0	–	–	–	–	149,530	10.1	6.0	12.2	6.2
	Thyroid	12,004	8.8	5.0	9.7	4.7	3,149	12.4	7.1	14.4	7.4	8,855	7.5	4.3	8.1	3.7
	Brain	10,158	8.2	5.4	10.6	5.2	5,959	8.5	4.9	10.9	6.0	4,199	7.8	5.9	10.1	4.1
	Prostate	123,508	8.0	5.1	10.4	5.4	123,508	8.0	5.1	10.4	5.4	0				
	Hodgkin's lymphoma	4,161	5.5	4.6	6.3	1.7	2,417	6.0	4.9	5.6	0.7	1,744	4.8	4.3	7.3	3.0
	Cervix uteri	8,798	4.8	2.9	5.8	2.9	0					8,798	4.8	2.9	5.8	2.9
	Testis	4,550	3.4	2.9	4.0	1.1	4,550	3.4	2.9	4.0	1.1	0				

**Fig. 1:**
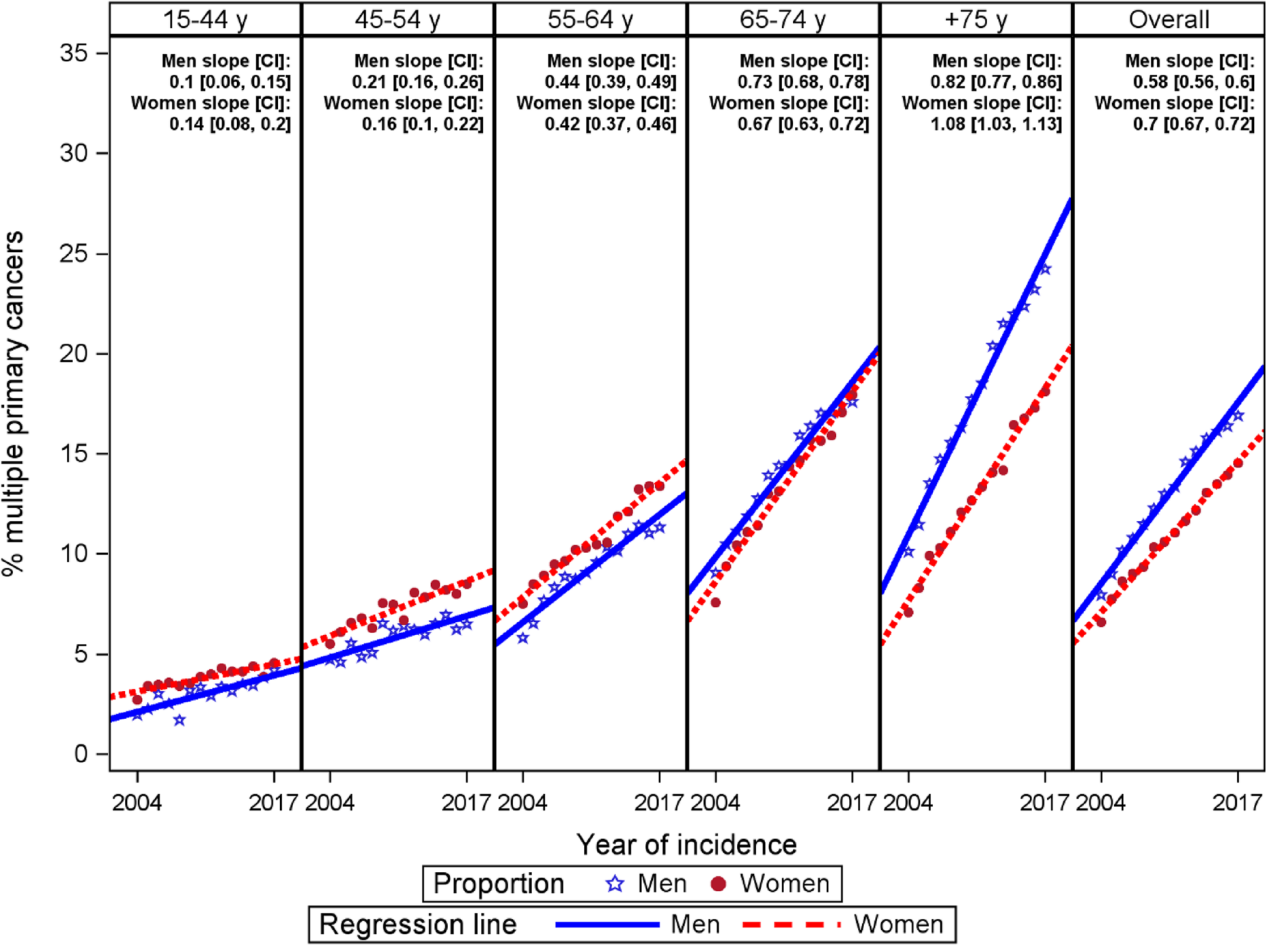
Proportion of multiple primary cancer (MPC), average increase in proportion of MPC by incidence year (slope) expressed in percentage point, its 95% confidence interval (CI) and corresponding regression line by gender and age category

The proportion of MPC as a function of incidence year, stratified by gender and age at diagnosis is presented in Fig. 1. A linear time trend for the MPC fraction can be observed for the age categories considered, therefore a simple linear regression model was applied to model the proportion of MPC over the incidence period for each age category. For all age categories combined, the proportion of MPC increases with 0.70 (CI: [0.67, 0.72]) percentage point per year for men, and with 0.58 (CI: [0.56, 0.60]) percentage point per year for women. The average increase by incidence year was higher with older age, ranging from 0.14 for men and 0.10 for women in the youngest age category (15–44 years) to respectively 1.08 and 0.82 percentage point in the oldest age category (≥ 75 years). For the age category ≥ 75 years and for all age categories combined, the slope seems to be higher for men than for women. Average increases for men and women in other age categories seem to be relatively similar.

### Impact of MPC on RS estimate

**Fig. 2:**
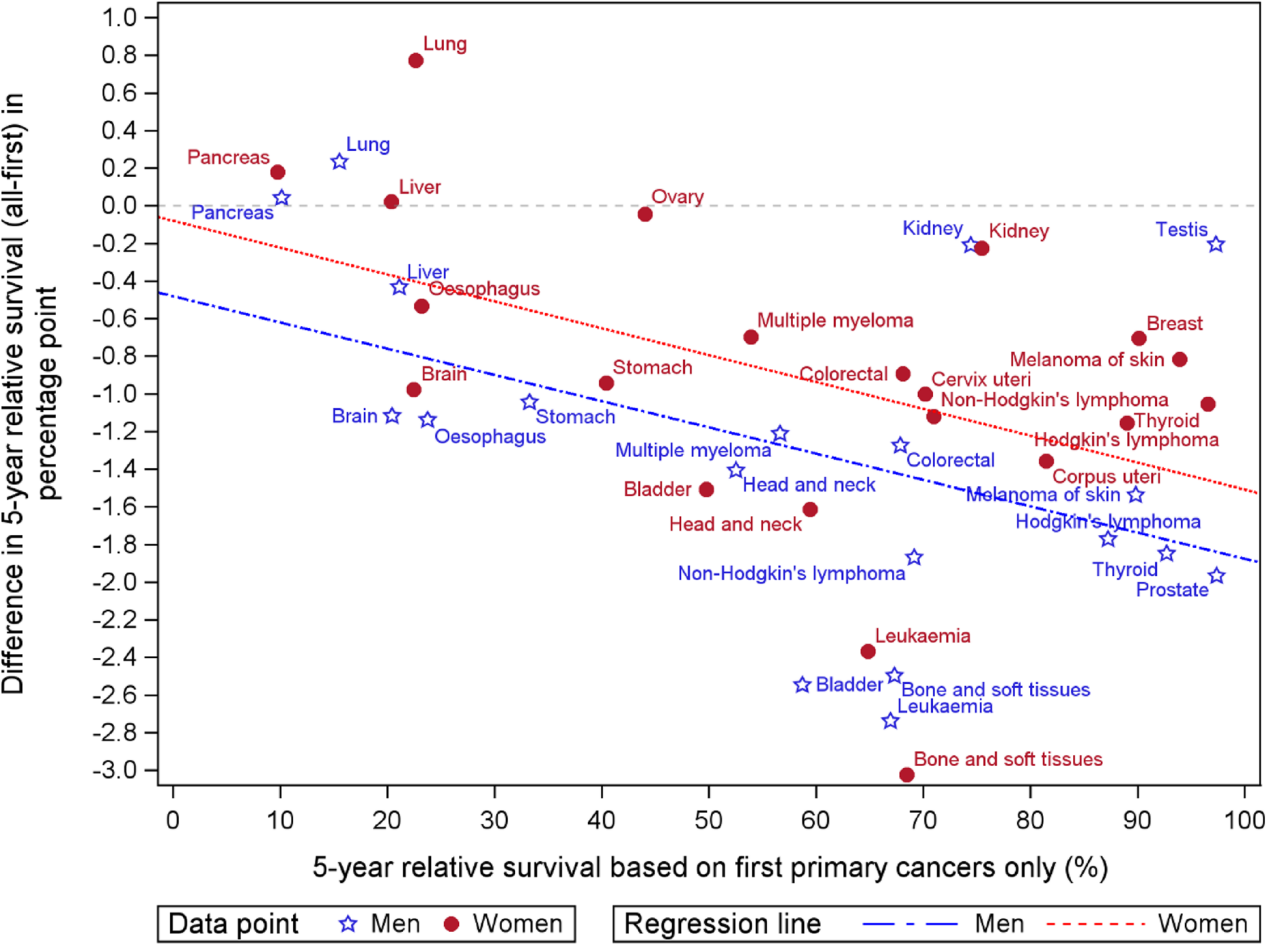
Difference between the 5-year relative survival (RS) calculated on all primary cancers and the 5-year RS calculated based on first primary cancer only according to the 5-year relative survival calculated on first primary cancer only by cancer site and gender and simple linear regression line over cancer type for men and women separately

The inclusion of MPC in the survival analysis of all cases resulted in a 0.7 percentage point lower value in 5-year crude RS for all cancers combined in men and 0.4 percentage point in women. The difference in 5-year RS probability when including MPC versus FPC only per cancer type and stratified by gender is given in Fig. 2. Except for lung and pancreas in men and women and liver in women, a lower RS proportion is observed when MPC were included in the analysis. The slopes of the two regression lines (-0.14, CI = [-0.27, -0.00] percentage point difference in men and -0.14, CI = [-0.28, -0.01] in women) are significantly negative at the 5% confidence level showing that the difference between the two methods becomes more negative when RS calculated based on FPC only increases. Similar results have been observed for age-standardised RS (data not shown). Difference value by gender and FPC site can be found in Supplementary file 3.

### Standardised incidence ratio (risk of developing a SPC)

**Fig. 3:**
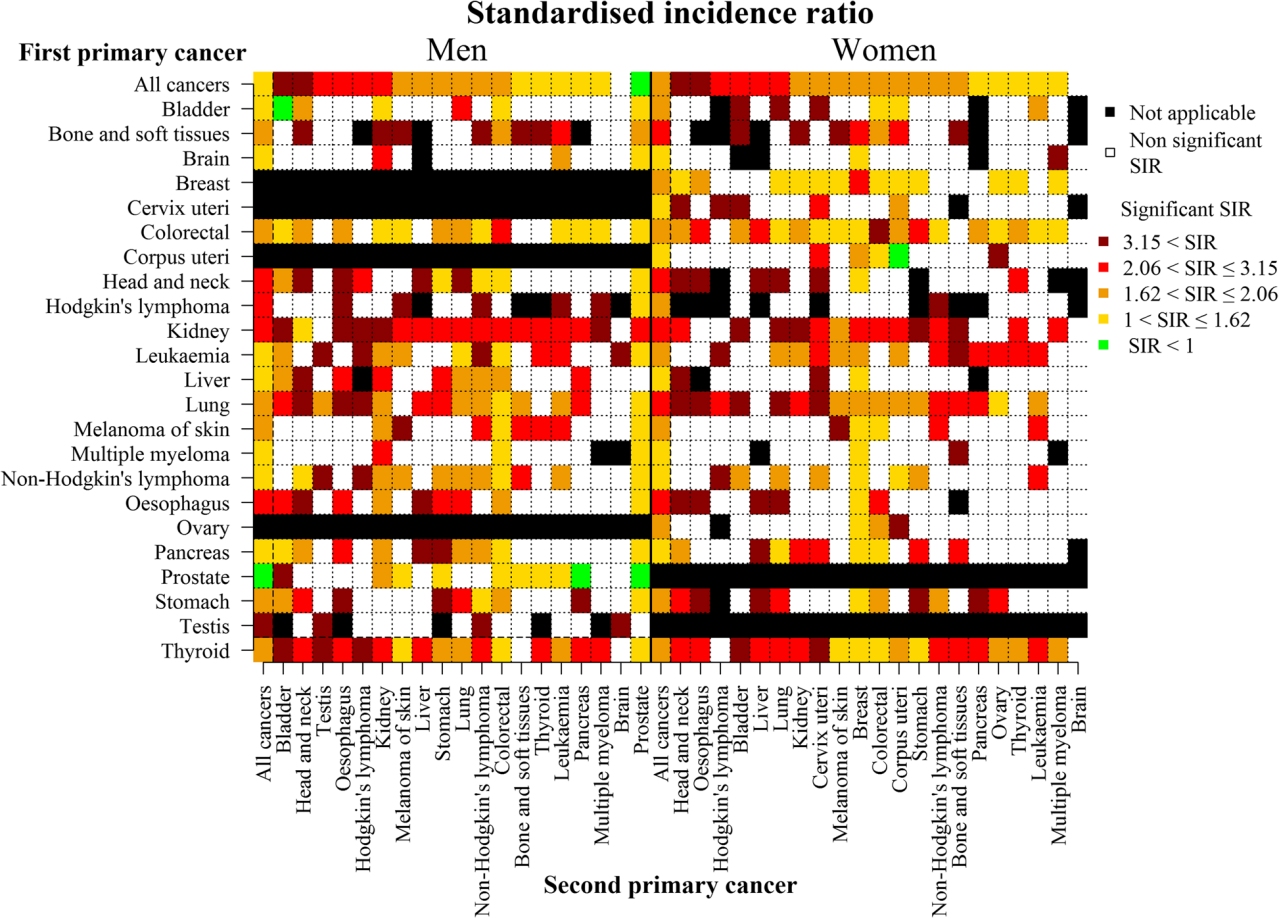
Standardised incidence ratio (SIR) of second primary cancer according to the cancer site of the first primary cancer and its of second primary cancer. SIRs for all cancers (on y axis) are classified by their value on x axis. The thresholds of SIR significantly higher than 1 have been defined according to quartiles of their values. The 5% cut-off on that many values can result in false positive or false negative classification

Overall, women with a FPC have 1.83 (CI: [1.81, 1.85]) times higher risk to develop a SPC than the risk for a FPC in the population without a previous cancer history. For men, this risk is 1.53 (CI: [1.55, 1.63]) times higher. The standardised incidence ratios (SIRs) as function of FPC and SPC by cancer site are presented in Fig. 3. The value of the SIRs and their CI can be found in the Supplementary files 4 and 5. The SIR to develop any SPC as function of the FPC tumour site is given in the left column in Fig. 3, per gender. All SPC site-specific SIRs were significantly higher than 1 except for prostate (SIR = 0.9, CI: [0.88, 0.92]) in men. The highest risks to develop a SPC compared to the population free of cancer were observed for male patients with testis (SIR = 3.98, CI: [3.29, 4.76]), kidney (SIR = 2.82, CI: [2.69, 2.95]) and thyroid (SIR = 2.78, CI: [2.43, 3.14]) as FPC. In women, highest overall SIRs were observed for kidney (SIR = 2.72, CI: [2.54, 2.92]), oesophagus (SIR = 2.23, CI: [1.99, 2.5]) and bone and soft tissues (SIR = 2.19, CI: [1.87, 2.54]) as FPC. The top line in Fig. 3 gives the SIR to develop a SPC at a specific site among the FPC population. All these overall SIRs are significantly higher than 1 except for brain in both men (SIR = 1.20, CI: [0.97, 1.46]) and women (SIR = 1.09, CI: [0.83, 1.42]) and for prostate (SIR = 0.96, CI: [0.94, 0.97]). In men, patients with a FPC have the highest risk to develop bladder (SIR = 3.71, CI: [3.6, 3.82]), head and neck (SIR = 3.51, CI: [3.4, 3.62]) and oesophagus (SIR = 2.8, CI: [2.95, 3.01]) as SPC compared to the population free of cancer. In women, highest overall SIRs are observed for head and neck (SIR = 3.57, CI: [3.35, 3.8]), oesophagus (SIR = 3.31, CI: [2.86, 3.8]), liver (SIR = 2.26, CI: [1.85, 2.75]) as SPC.

Some specific reciprocal relationships can be observed between cancer sites. For example, in men and women, patients with head and neck cancer as FPC have a higher risk to develop a lung cancer as SPC (SIR = 3.98, CI: [3.55, 4.45] in men and SIR = 6.04, CI: [4.66, 7.77] in women) and patients with lung cancer as FPC have a higher risk to develop a head and neck cancer as SPC (SIR = 7.87, CI: [7.47, 8.28] in men and SIR = 12.4, CI: [11.01, 13.9] in women) compared to the population free of cancer. Bi-directional relationships were also found between oesophagus and lung, head and neck and oesophagus, Hodgkin’s lymphoma and non-Hodgkin’s lymphoma, between ovary and corpus uteri or between bladder and prostate. SIR for a SPC at the same site are also presented (Fig. 3), however, comparisons and interpretation of results must be carefully done because it depends of the cancer sites and the definition of MPC that were used.

### Difference in stage distribution between FPC and SPC

**Fig. 4:**
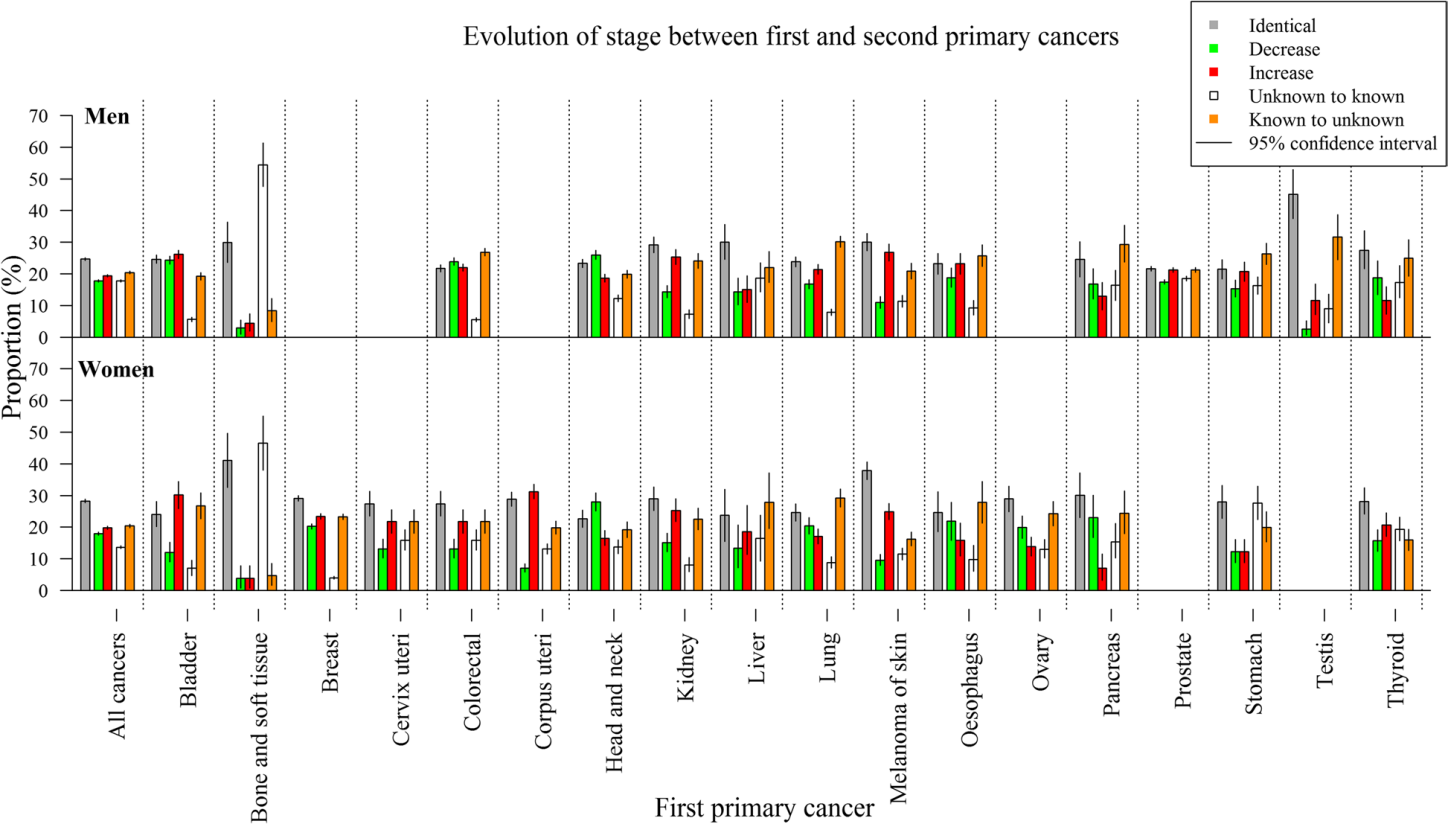
Proportion of scenarios of the evolution of combined stage from first primary cancer to second primary cancer and their 95% confidence interval (CI) by cancer site and gender. First and second primary cancer diagnosed the same day are excluded of this analysis

The proportion of identical stages for FPC and SPC is 24.7% (CI: [24.2, 25.1]) in men and 28.2% (CI: [27.7, 28.8]) in women. The FPC is slightly more often diagnosed at a higher stage compared to the SPC (19.5%, CI: [19, 19.8] in men and 19.8%, CI: [19.3, 20.3] in women) than it is diagnosed at a lower stage (17.8%, CI: [17.4, 18.2] and 17.9%, CI: [17.4, 18.4], respectively). We also found slightly more patients switching from a known stage to an unknown stage (20.4%, CI: [20, 20.8] in men and 20.4%, CI: [19.9, 20.9] in women) than the contrary (17.7%, CI: [17.3, 18.1] in men and 13.6%, CI: [13.2, 14.1] in women). This is also the case at cancer site level for the majority of FPC. Some exceptions can be observed in both genders. In patients with bone and soft tissue as FPC, the proportion of patients switching from an unknown to a known stage is high compared to other cancer sites. The same observation (as for head and neck) can be done (to a lesser extent in men) for pancreas FPC. The proportions of different defined situations as function of gender and FPC site are presented in Fig. 4. Proportions values by gender and FPC site and their CI can be found in Supplementary files 6 and 7.

## Discussion

Improvements in screening, (early) diagnosis, treatment and follow-up resulted in increased survival of cancer patients [1, 2]. Together with an overall increasing life expectancy, these improvements have increased the number of patients who present with a subsequent primary cancer. Here, we have analysed for the first time the MPC present in the database of the Belgian Cancer Registry, incidence period 2004–2017. The percentage of MPC during the whole period was estimated to be 12.2% and increased linearly since 2004, the onset of nationwide cancer registration. The overall percentage of MPC could still increase in years following the considered study period if cancer survival still increases, and because the correct identification of MPC as MPC (and not as FPC) increases with registration time. A previous study suggested that the difference in proportion of MPC between cancer registries depends on registration time for registries operating for less than 10 years [8] (proportion of MPC of registries increasing with registration time), suggesting that the registration effect is probably limited after 10 years. No link between register time and proportion of MPC was observed for older cancer registries. A review study considering data from registries situated in North America, Europe and Oceania found that the proportion of MPC ranged between 2 and 17% [22]. Rosso et al. [8] found a range between 0.7% and 12.9% among European cancer registries. Our current estimate of 12.2% is within these ranges. However, many factors can influence this estimation such as the applied definition of primary cancers, the follow-up time available from the FPC, the population under study [23], but also the considered period as cancer survival has increased over the years. Proportion of MPC depends on cancer site, gender and region. Brenner et al. [9] found similar distribution of MPC according to cancer site. Among MPC, Rosso et al. [8] found that colon, lung, breast and prostate are the most frequent sites which is consistent with results from our study. In Belgium, the proportion of MPC is higher in males than in females. In Switzerland, a higher proportion of SPC was also observed in males than in females [7]. In our study, the higher proportion of MPC in Flanders is explained by a longer registration history compared to the other regions. If we consider only information from 2004 to define the multiple status of primary cancers, proportions of MPC are similar for the different regions (data not shown).

This work shows that the MPC proportion depends also on the age at diagnosis of patients. Indeed, the proportion of MPC increases faster over incidence years for higher age. This difference may be explained by the fact that MPC were mainly diagnosed in older individuals, despite of the recent onset at the Belgian Cancer Registry (2004) and by consequence a short period of study. Indeed, age is itself one of the most important risk factor of cancer development [24].

For all cancer sites combined, during the period 2004–2017, the estimated 5-year RS decreased with 0.6 percentage point when including MPC. The overall impact of MPC on RS is consistent with observations by Rosso et al. [8] and Ellison et al. [10], who found a difference of -0.6 and -0.9 percentage point, respectively (with a range between 0 and -2.2 according to the considered registries in Rosso et al. [8]. We also found a larger impact of MPC on 5-year RS for cancer sites with better survival. For pancreas and lung cancer in men and women and liver cancer in women, a higher 5-year RS was observed when MPC are included. A possible explanation for this increase is that, more early stages are observed in MPC than in FPC for these two cancer sites (data not shown). Rosso et al [8]. found a relationship between the proportion of MPC and the impact of MPC on RS estimates, with a higher impact for registries with the highest proportions.

The relative survival estimates reported in this study are with or without inclusion of multiple primary cancer registrations. The percentage of MPC varies from 3 to 18% according to cancer site (see Table 1). Reporting relative survival for a cohort of MPC only can be challenging. It is indeed likely that individuals with a given combination of comorbidities or risk factors (smoking, alcohol consumption, high BMI) can be at higher risk for specific multiple primaries (head and neck cancers, lung cancer, liver, …). Relative survival estimates for a cohort for which the comorbidity pattern is markedly different compared to the general population can be expected to be biased, i.e. cancer mortality would be overestimated. 

In several countries [3–7], patients with a FPC were found to have a higher risk of developing a subsequent primary cancer compared to the population without cancer history. In our study, we found that the overall SIR in Belgium was 1.64 (CI: [1.63, 1.66]). In other words, having a FPC increased the risk of cancer by more than 50%, compared to the population free of cancer. Differences in study design and selection criteria (i.e., inclusion of synchronous cancers, the time to consider cancers as synchronous and/or patients with SPC at the same (sub)site than the FPC) make comparison of results difficult. For example, in France (SIR = 1.36), patients who have the two FPC diagnosed within 2 months have been excluded and the study focuses on the risk of SPC occurring in a different subsite as the FPC [3]. A similar rule concerning synchronous cancers have been applied in the USA [4] (SIR = 1.14) and in Italy [5] (SIR = 0.93 when synchronous cancers are excluded, and SIR = 1.08 when synchronous cancers are included). In a Japanese study, cancers diagnosed at the same site were excluded (SIR = 1.10) [6]. In Switzerland, SPC have been defined as subsequent primary cancer occurring at least 6 months after the first cancer (SIR = 1.2 in women and SIR = 1.18 in men) [7]. In our study, we conducted a sensitivity analysis, excluding patients with the two FPC diagnosed within 2 months (< 61 days between FPC and SPC), resulting in a SIR equal to 1.27 with CI: [1.26, 1.28] (SIR = 1.24 CI: [1.23, 1.26] in men; SIR = 1.32 CI: [1.30, 1.33] in women). As in the Italian study [5], a decrease in SIR is observed when patients with synchronous cancers (within 2 months) are excluded. In studies that calculated SIRs by time interval [5, 6], the SIR was highest for time interval(s) following diagnosis and decreased thereafter. It could be a consequence of intensive investigations in patients attending diagnosis and treatment for a FPC. Excluding patients with SPC at the same site than the FPC can considerably impact the estimates of SIRs [25]. In our study, the SIRs at FPC or SPC level is not markedly different when removing patients with same-site FPC and SPC among women, and is only modified for prostate and testis as FPC in men (data not shown). For testis, the SIR decreases from 3.98 (CI: [3.29, 4.76]) to 1.48 (CI: [1.56, 2.03]) when patients with an SPC at the same site are excluded. This shows that the higher risk to develop a new cancer for testis cancer patients compared to population free of cancer is for a large part due to a high risk to develop a SPC at the same site. On the contrary, for prostate cancer, the SIR significantly lower than 1 (SIR = 0.9, CI: [0.88, 0.92]) becomes significantly higher than 1 (SIR = 1.73, CI: [1.69, 1.77]) when patients with FPC and SPC at same site are excluded. The lower risk to develop a SPC for prostate cancer patients compared to population free of cancer is by consequence mainly due to the very low risk to develop a SPC at prostate. It may be due in part to radical prostatectomy which is a frequent treatment [26, 27]. Another potential explanation is that older age prostate cancer survivors show a high prevalence of suboptimal health behaviors [28] which could be higher than in the corresponding population free of cancer. Patients with corpus uteri as FPC, cancer for which hysterectomy is a standard treatment [29], have also a lower risk to develop a SPC at the same site compared to the population free of cancer (SIR = 0.38, CI: [0.24, 0.58]). However, excluding patients with the two FPC at the same site has a little impact on the SIR (SIR = 1.41, CI: [1.33, 1.5] with all patients and SIR = 1.49, CI: [1.41, 1.58] when patients with FPC and SPC at same site are excluded).

We found some bi-directional relationships such as between cancers of the oesophagus, lung and head and neck, these links have been also found in other studies [3, 4, 7]. This may be explained by the fact that these cancer sites share strong etiological factors and typically occur in a population with smoking and drinking habits [30, 31]. A bi-directional link has also been found between corpus uteri and breast and between ovary and corpus uteri, which share hormonal, genetic and etiologic factors [32–34]. These associations have also been found in Taiwan and were strongest within the first 5 years after the FPC [35].

This study presents non adjusted results for the risk of a SPC. However, it would be interesting for a future work to use a Poisson Model in order to explore more in depth the association between patient characteristics and risk of a SPC.

We found more patients with a higher stage for the FPC compared to the SPC than a lower stage. This finding is rather surprising because patients with a FPC are likely to be monitored more intensely than the population free of cancer and subsequent cancers can thus be expected to be diagnosed at earlier stage. However, SPC could be associated with cancer sites that are typically diagnosed at an advanced stage. In this way, among SPC, we found more lung cancers (difference of 10 percentage point) and more pancreatic cancers (difference of 2 percentage point) than among FPC. On the contrary, we found less prostate cancers (difference of 8 percentage point) and breast cancer (difference if 4 percentage point) among SPC. We conducted a sensitivity analysis, keeping only patients with their FPC and SPC at the same site, resulting in a higher proportion with lower stage than higher stage in both genders (respectively, 22.3%, CI: [20.8%, 23.8%] and 17.3%, CI: [16%, 18.7%] in men and 26.5%, CI: [25.4%, 27.8%] and 14.6%, CI: [13.7%, 15.6%] in women). A higher proportion of patients switching from a known stage to an unknown stage is observed than vice versa. Potentially, a very poor prognosis could limit further clinical examination of the SPC, resulting in an unknown stage. The registration quality in Belgium has improved over time, reducing the proportion of unknown stages year after year, which could influence the proportion of patients switching from a known stage to an unknown stage depending on the incidence year of the SPC. In patients with bone and soft tissue as FPC, the proportion of patients switching from an unknown to a known stage are high compared to other cancer sites which is explained by the limited use of TNM for those cancer types.

The use of stage to sort primary cancers diagnosed the same day allows to consider all patients with at least two primary cancer diagnoses as patients with MPC and to include all primary cancers in these analyses. This criterion is clear and easy to use. Even though the speed at which the disease progresses could be different for several cancers diagnosed the same day, most of the time, cancer in more advanced stage is the most important in decision making about treatment and follow-up. Moreover, another methodological aspect must be pointed out. The identification of MPC depends on the rules which are applied. For example, SEER rules (Surveillance, Epidemiology, and End Results Program) are more complex and classify more primary cancers as MPC than IARC rules [36, 37]. A limitation of this study is the short complete registration time available at the Belgian Cancer Registry. Indeed, the risk that a MPC is registered wrongly as FPC could be greater compared to registries with longer registration time and impact the different results presented in this article. Moreover, MPC of previous residents of Belgium who are diagnosed abroad are not registered at the Belgian Cancer Registry. The results presented here depend also on the study period and probably will change in the future.

## Conclusions

In conclusion, after 14 years of cancer registration in Belgium, this study described for the first time MPC from different angles of interest. The proportion of MPC depends on age, cancer site and gender. This proportion has linearly increased from the start of complete registration in 2004. The inclusion of MPC impacts RS estimates and must be taken into account in order to make fair comparisons. As in other countries, Belgian cancer survivors have an increased risk to develop an SPC compared to the population without a history of cancer.

## Supplementary Information

**Additional file 1. ** Registration rules for multiple primary cancers applied.

**Additional file 2.** Median time between first and second primary tumour registration, in years, incidence period 2004-2017.

**Additional file 3.** 5-year relative survival according to inclusion or non-inclusion of multiple primary cancers.

**Additional file 4.** Standardised incidence ratios for a second primary cancer in men.

**Additional file 5.** Standardised incidence ratios for a second primary cancer in women.

**Additional file 6.** Evolution of combined stage from first primary cancer to second primary cancer in men.

**Additional file 7.** Evolution of combined stage from first primary cancer to second primary cancer in women.

## Data Availability

The datasets generated and/or analysed during the current study are not publicly available due to privacy reasons but are available from the corresponding author on reasonable request. The pseudonymized data can be provided within the secured environment of the Belgian Cancer Registry according to its regulations, and only upon approval by the Information Security Committee.

## Abbreviations

FPC: First primary cancer
IARC: International Agency for Research on Cancer
ICD-O: International Classification of Diseases for Oncology, Third Edition
MPC: Multiple primary Tumours
RS: Relative Survival
SIR: Standardised incidence ratio
SPC: Second primary Tumours
