# Supplementary material for: The epidemiology of multiple primary cancers in Belgium (2004–2017): Incidence, proportion, risk, stage and impact on relative survival estimates
Source: BMC Cancer. 2023 Apr 17;23:349. doi: 10.1186/s12885-023-10777-7 (PMC10108509; doi:10.1186/s12885-023-10777-7)

Rules applied at Belgian Cancer Registry for registration of multiple primary cancers based on topography, laterality, histology and behavior

Concerning the topography, the general rule states that one tumour can be registered in each organ (which are defined with the first two digits of the ICD-O code except for some organs like the colon, bones and skin, in which the whole ICD-O code refers to one separate organ). Moreover, paired organs must be seen as different organs. Two different tumours must thus be registered in case of bilateral tumours.

Nevertheless, sometimes, multiple tumours in the same organ have to be registered. To be able to take that decision, we use the Modified groups of Berg, a tool that groups together the closely related histological codes. Lesions belonging to the same group of Berg are morphologically identical or very similar, and are therefore considered as the same tumour. Lesions belonging to different groups of Berg are morphologically unrelated and are considered as different tumours, and therefore registered distinctly.

Finally, for tumours located in the same organ and that are morphologically related, the behaviour of the tumour (and sometimes the incidence date) is also important to determine what should be registered. Different situations must be considered:

- Situation 1: if 2 different tumours with the same behaviour then only 1 tumour will be registered (in this case, the second tumour is considered as a relapse/recurrence of the first tumour. The period between both tumours has no influence).

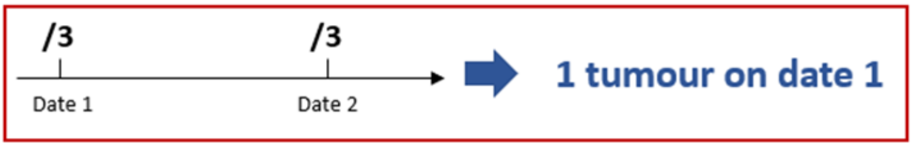

- Situation 2: if one in situ tumour followed by one invasive tumour with more than 3 months between the incidence dates then 2 different tumours should be registered (in this case, the second tumour will not be considered as a relapse of the first one since the behaviour gets worse).

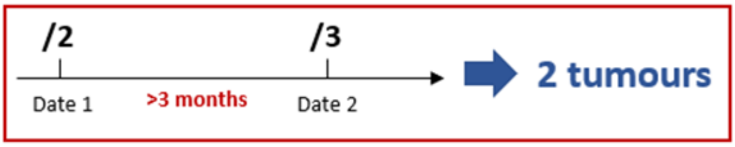

• Situation 3: if one in situ tumour followed by one invasive tumour with less than 3 months between the incidence dates then only 1 tumour should be registered (in this case, we assume that the invasive component was already present but missed during the first diagnosis. Since we consider the diagnosis period as being 3 months, such a case will be considered and registered as only one tumour with different foci/components).

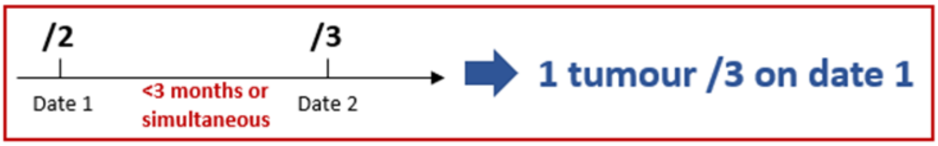

• Situation 4: if one invasive tumour followed later by one in situ tumour then only 1 tumour should be registered (in this case, the second tumour is considered as a relapse of the first one for the registration, regardless the time period between both).

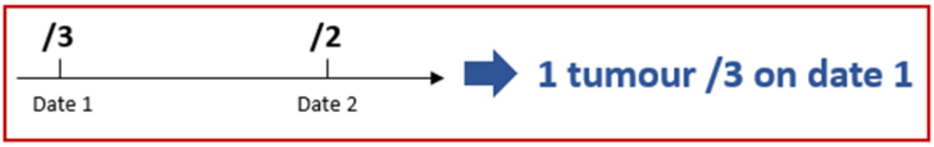

Supplement: Supplementary file 1 — Additional file 1. Registration rules for multiple primary cancers applied. [file 12885_2023_10777_MOESM1_ESM.pdf]
